# The cross-sectional area of the vagus nerve is not reduced in Parkinson's disease patients^☆^

**DOI:** 10.1016/j.ensci.2022.100400

**Published:** 2022-05-05

**Authors:** Laura C.J. Sijben, Werner H. Mess, Uwe Walter, A. Miranda L. Janssen, Mark L. Kuijf, Mayke Oosterloo, Wim E.J.. Weber, Marcus L.F. Janssen

**Affiliations:** a Department of Clinical Neurophysiology, Maastricht University Medical Center, Maastricht, the Netherlands; b School for Mental Health and Neuroscience, Faculty of Health, Medicine and Life Sciences, Maastricht University, Maastricht, the Netherlands; c Department of Neurology, Rostock University Medical Center, Rostock, Germany; d German Centre for Neurodegenerative Diseases (DZNE), Rostock, Germany; e Department of Methodology and Statistics, CAPHRI Care and Public Health Research Institute, Maastricht University, the Netherlands; f Department of Neurology, Maastricht University Medical Center, Maastricht, the Netherlands

**Keywords:** Parkinson's disease, Vagus nerve, Ultrasonography, Cross-sectional area, Autonomic symptoms

## Abstract

**Background:**

Recent studies have revealed the importance of the gut brain axis in the development of Parkinson's disease (PD). It has also been suggested that the cross-sectional area (CSA) of the vagus nerve can be used in the diagnosis of PD. Here, we hypothesize that the CSA of the vagus nerve is decreased in PD patients compared to control participants.

**Methods:**

In this study we measured the CSA of the vagus nerve on both sides in 31 patients with PD and 51 healthy controls at the level of the common carotid artery using high-resolution ultrasound.

**Results:**

The mean CSA of the left vagus nerve in the PD and the control group was respectively 2.10 and 1.90 and of the right respectively 2.54 and 2.24 mm2. There is no difference in CSA of the vagus nerve in PD patients compared to controls (*p* = .079). The mean CSA of the right vagus nerve was significantly larger than the left (*p* < .001). Age, sex and autonomic symptoms were no significant predictors of the CSA of the vagus nerve.

**Conclusion:**

These findings show that the CSA of the vagus nerve using ultrasonography is not a reliable diagnostic tool in the diagnosis of PD.

## Highlights

• There is no difference in cross-sectional area of the vagus nerve in Parkinson disease compared to controls.
• Autonomic symptoms are not related to the cross sectional are of the vagus nerve.
• Measurement of the vagus nerve cross-sectional area has a high inter-observer correlation.

## 1 Introduction

Parkinson's disease (PD) is a progressive neurodegenerative disorder resulting in a clinical hypokinetic rigid syndrome. Besides the classical motor symptoms, PD is associated with non-motor symptoms. Among these are autonomic symptoms, such as orthostatic hypotension, constipation and urinary incontinence. Autonomic symptoms are frequently reported by PD patients and have been suggested as preclinical symptoms [1].

The main neuropathologic hallmark is the formation of cytoplasmic inclusions called α-synuclein-enriched Lewy bodies and Lewy neurites. These α-synuclein aggregations are found throughout the brain, most pronounced in the substantia nigra [2,3]. Neural cell loss is most abundant in the substantia nigra compacta. Interestingly, the pathological α-synuclein is also present in the peripheral nervous system, more specifically in the vagus nerve, which plays an important role in autonomic control [4,5]. Braak and colleagues hypothesized that a neurotropic pathogen entering the brain may be involved in the pathology of PD [5,6]. This may occur via nasal or gastric route, causing transsynaptic cell-to-cell, prion like, transmission of α-synuclein [5,6]. It is suggested that the pathogen invades the brain by entering the nasal mucosa, followed by the olfactory bulb and the anterior olfactory nucleus into the olfactory structures of the temporal lobe. The gastric route starts in the enteric nervous system, where α-synuclein enters the Meissner's and Auerbach's plexus. From that point α-synuclein is transported to the lower part of the brainstem through the vagus nerve. α-synuclein spreads from the vagus dorsal motor nucleus into the medulla and the rest of the brain [6]. A recent study in an animal model supports the hypothesis that the propagation of pathologic α-synuclein via vagus nerve causes PD [7].

In line with these findings, it has been shown that a vagotomy or appendectomy early in life are associated with a lower risk to develop PD later in life [[8], [9], [10]] [[11], [12], [13], [14]]. The appendix appears to be the largest source of α-synuclein, whereas the vagus nerve only seems to be the transporter [11,15]. Based on these findings it has been postulated that the cross-sectional area (CSA) of the vagus nerve decreases in PD. In fact, several studies have been published showing a significant decrease in the CSA of the vagus nerve in PD [[16], [17], [18], [19]]. It has even been hypothesized that atrophy of the vagus nerve already occurs at a pre-clinical stage [17]. These findings highlight the potential to use the assessment of the CSA of the vagus nerve rostral to the carotid bifurcation by using B-mode ultrasound as a fast and low-cost diagnostic test. Moreover, this method potentially identifies persons at risk even before a clinical diagnosis of PD can be confirmed. It is however important to note that two studies using B-mode ultrasound could not confirm that the CSA of the vagus nerve was decreased in PD [20,21].

The primary aim of this study is to assess if the CSA is decreased in PD. We hypothesize that the CSA of the vagus nerve is reduced in PD patients compared to controls using ultrasonography. Our secondary aim is to assess if the CSA correlates with autonomic symptoms in PD. We will also assess the interrater reliability and compare two different methods to determine the CSA. To these aims, we conducted a cross-sectional study in which we acquired the CSA of the left and right vagus nerve using ultrasonography. Autonomic symptoms were assessed by a standardized questionnaire.

## 2 Material and methods

The study was given a positive advice by the local ethics committee and all participants gave written informed consent before participation (*METC Maastricht University Medical Center, the Netherlands, protocol number 2019–1223*). All participants underwent a B-mode ultrasound examination of the vagus nerve and filled in a short questionnaire about autonomic complaints (Scales for Outcomes in Parkinson's Disease - Autonomic Dysfunction; SCOPA-AUT).

### 2.1 Participants

The inclusion criterion was a clinical diagnosis of PD made by the treating neurologist in accordance with the UK Brain Bank criteria for the PD group [22]. Diagnose was at least three years ago and a good levodopa response was observed. Persons with any red flags that might suggest another neuropathology were excluded. The control group consisted of persons without PD. Exclusion criteria for both groups were other neurodegenerative disorder(s) and previous surgery in proximity of the vagus nerve (for example carotid endarterectomy or implantation of vagus nerve stimulator). Participants were recruited by their neurologist. The controls were recruited at the clinic from patients who were referred for carotid artery ultrasound.

### 2.2 Ultrasound

**Fig. 1:**
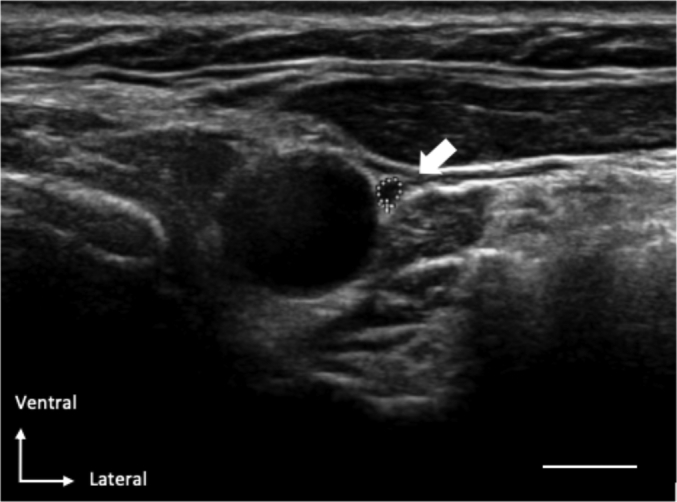
Representative example of an ultrasound image in which the vagus nerve (dotted circle) is positioned ventrolateral to the common carotid artery. The scale bar equals 0.5 cm.

Ultrasound of the vagus nerve was performed by two experienced nerve sonographers (CR, NG) and one less experienced sonographer (LS) using the Philips IU22 with a linear 17.5 MHz transducer. The sonographer was not blinded for the participant's identity, being a patient or a control. The vagus nerve was visualized in a coronal plane anterolateral to the common carotid artery (Fig. 1). The vagus nerve CSA was measured on site during the scanning session by outlining the inner side of the hyperechoic epineural rim using the trace function of the ultrasound machine. At each side the CSA of the vagus nerve was measured twice. The mean of the two measurements was used for statistical analyses.

To ensure a good interrater reliability, the contour of the vagus nerve within the hyperechoic epineural rim was outlined by two independent sonographers (first by one less experienced (LS) and next offline on the acquired images by an experienced and completely blinded sonographer (CR) on the acquired ultrasound images in ten participants.

To asses possible differences between our method applied (as described above) and one of the earlier studies [19], we measured the longest cross-sectional diameter *a* and the diameter *b* perpendicular to *a* (as described by Walter et al.) in 34 participants. The CSA was calculated using the following formula: (a*b*π)/4. The diameters were measured at the same place as the contour of the vagus nerve was measured. This also happened immediately during the ultrasound.

### 2.3 Questionnaire

On the same day as the ultrasound examination took place all participants filled in the SCOPA-AUT, a validated questionnaire about autonomic complaints [23,24]. The SCOPA-AUT consists of six domains: gastrointestinal, urinary, cardiovascular, thermoregulatory, pupillomotor and sexual functioning. There are different numbers of questions for each domain, with a total of 23 questions. For each question a score from 0 to 3 points can be obtained, the maximum score that can be obtained is 69 points. The total sum score was used for further analyses.

### 2.4 Statistics analysis

Baseline characteristics from the PD and control group were compared with an independent-samples *t*-test for age and the result on the SCOPA-AUT and with a Chi-square test for sex. All subjects were measured twice, left and right vagus nerve. To account for the dependence among the 2 repeated measurements per subject marginalized multilevel model (MMM) analyses were performed. To select the most appropriate covariance structure (an unstructured matrix or a compound symmetry matrix), the restricted maximum likelihood ratio test was used. Firstly, a MMM analysis including group (PD patients and controls), side (left and right) and their interaction was conducted to estimate the difference between the two groups. Secondly, possible clinical and demographic confounders were tested one by one in an univariable model. Finally, a multiple MMM analysis was performed including group, side and all possible confounders. The final model was constructed by backward elimination of non-significant variables with the exception of the group variable.

The Intraclass Correlation Coefficient (ICC (2,1) classification according to Shrout and Fleiss) was calculated to compare the two different types of measurements for the CSA and to compare the observations by the two independent sonographers [25]. The SPSS statistical computer package (version IBM SPSS Statistics for Macintosh, version 25 (IBM Corp., Armonk, N.Y., USA)) was used for all statistical analyses. A *p*-value threshold of <0.05 was used for all statistical analyses.

## 3 Results

### 3.1 Demographics (Table 1

We recruited 82 participants (31 PD and 51 controls) between December 2019 and April 2020 at the Departments of Neurology and Clinical Neurophysiology of Maastricht University Medical Center (MUMC+). The clinical and ultrasonographic findings are summarized in Table 1.

**Table 1:** **Demographics, clinical scores and ultrasonography of the Parkinson's and control group.** Values except gender are presented as means + SD. For gender number and percentage of females are given. CSA, cross-sectional area in mm^2^. SCOPA-AUT, Scales for Outcomes in Parkinson's Disease - Autonomic Dysfunction. *P*-values are from comparisons between PD patients and the control group using an independent-samples *t*-tests for age, sex and SCOPA-AUT and a repeated measurement ANOVA for the CSA of the left and right vagus nerve.

	Parkinson (*n* = 31)	Control (*n* = 51)	P
**Demographics**			
Age (years, M + SD)	69 (8)	72 (8)	0.262
Sex (female, N%)	15 (48%)	22 (43%)	0.643
Disease duration (months, M + SD)	95 (66)		
Hoehn & Yahr stage (stage N%, PD group)	Stage 1: 1 (3%) Stage 4: 4 (13%)	Stage 2: 22 (71%) Stage 5: 0	Stage 3: 4 (13%)
Side onset (N%, PD group)	Left: 14 (45%) Right: 16 (55%)		

**Clinical scores**			
SCOPA-AUT (M + SD)	16 (7)	13 (8)	0.078

**Ultrasonography** (CSA, mm^2^, M + SD)			
Left vagus nerve	2.10 (0.57)	1.90 (0.56)	0.391
Right vagus nerve	2.54 (0.70)	2.24 (0.68)	0.391

Males and females were evenly disturbed among groups. In the control group, nine (18%) participants had diabetes mellitus without complications, 31 (62%) participants had cardiovascular disease, and one (2%) had a polyneuropathy. The average number of medications the participants used at the time of the ultrasound was four (range: 0–11). In one participant the medical history was unavailable. In the Parkinson group, two (7%) patients had diabetes mellitus without complications, five (16%) patients had cardiovascular disease and none of the participants had a history of polyneuropathy. The average number of medications the participants used apart from their Parkinson medication at the time of the ultrasound was two (range: 0–14).

The mean total score on the SCOPA-AUT was 16 ± 7 in the PD and 13 ± 8 points in the control group. There was no significant difference between the total SCOPA-AUT score of the two groups (*p* = .078).

### 3.2 CSA of the vagus nerve

**Fig. 2:**
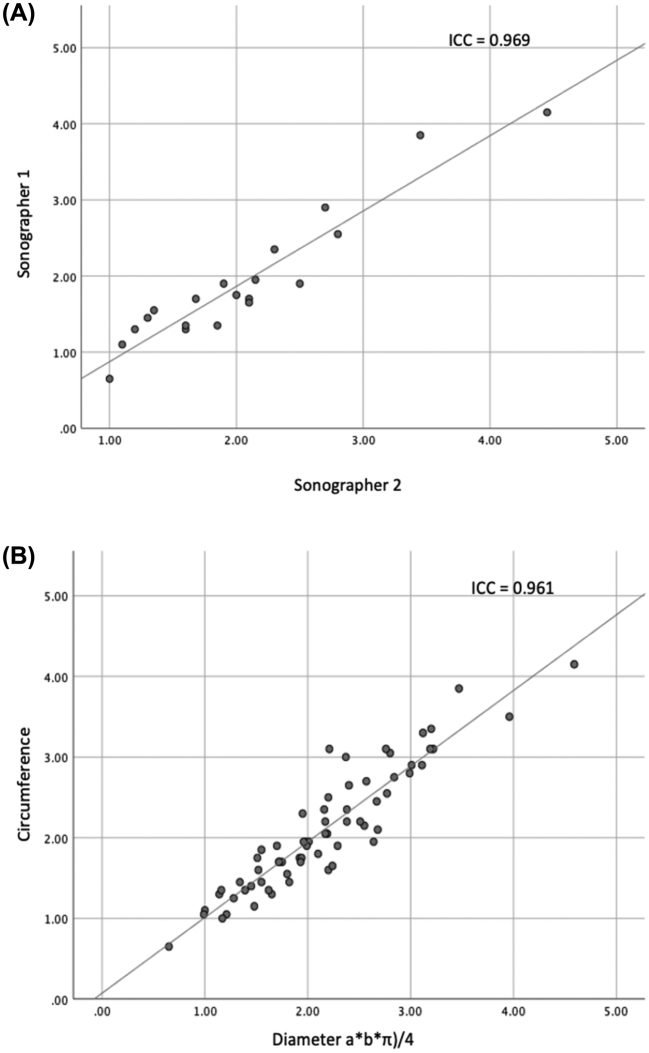
Scatterplots showing the linear correlation between A) the CSA determent by two independent sonographers, B) the different methods to obtain the CSA (respectively circumference on the y- and diameter a*b*π)/4 on the x-axis).

There was no difference in nerve echogenicity and no anatomical variants were observed between de PD group and controls. First, we assessed if a good interrater reliability was achieved. The ICC showed a strong agreement between the independent sonographers (ICC = 0.969, *p* < .001). (See figure 2A.) Secondly, we assessed if our investigation method was compatible with an earlier described method to estimate the CSA using the diameter of the vagal nerve in two directions [19]. The ICC showed a strong agreement between the two methods to estimate the CSA (ICC = 0.961, p < .001).

**Fig. 3:**
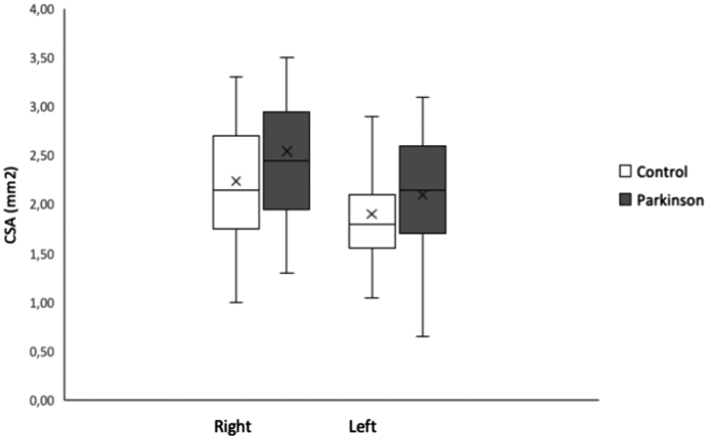
Barplots representing the mean of the CSA of the left and right vagus nerve of the Parkinson's disease and control group. Error bars represent standard deviation of the mean. CSA, cross-sectional area in mm^2^.

The mean CSA of the left vagus nerve in the PD and the control group was respectively 2.10 ± 0.57 mm^2^ and 1.90 ± 0.56 mm^2^ and of the right respectively 2.54 ± 0.70 mm^2^ and 2.24 ± 0.68 mm^2^ (Fig. 3). MMM analysis revealed no significant interaction between group and side (t(80) = 0.863, *p* = .391) and was removed from the model (Table 2). Furthermore, the MMM analysis determined that the mean CSA of the right vagus nerve was significantly larger than the left in both PD and controls t(81) = 6.544, *p* < .001, d = 0.379, 95%CI [0.264,0.495] (right-left). (See figure 2B.)

**Table 2:** Univariable and multivariable models.

	Estimate	SE	95% CI	P
Univariable Model				
Side (right)	0.379	0.058	0.264–0.495	<0.001
Group (PD)	0.223	0.125	−0.026–0.473	0.079
Sex (female)	−0.228	0.122	−0.470–0.015	0.066
Age	−0.009	0.008	−0.024–0.007	0.271
Diabetes Mellitus (yes)	0.197	0.179	−0.158–0.553	0.273
Cardiovascular disease (yes)	−0.098	0.124	−0.344–0.148	0.428
SCOPA-AUT	−0.003	0.008	−0.020–0.013	0.709

Multivariable Model				
Side (right)	0.379	0.059	0.262–0.496	<0.001
Group (PD)	0.218	0.122	−0.025–0.461	0.078
Sex (female)	−0.260	0.119	−0.497–−0.023	0.032

Univariable models; where the independent variables are compared with cross-sectional are of the left and right vagus nerve. Multivariable models including all the independent values with significant value.

### 3.3 Relationship between autonomic symptoms (and other variables) and CSA of the vagus nerve (Table 2)

Univariable MMM analyses including age, sex, group, diabetes mellitus, cardiovascular disease or SCOPA-AUT as independed variable showed no significant effects at a significance level of 0.05. Though the CSA difference between males and females was near significance (t(80) = −1.867, *p* = .066, d = −0.228, 95% CI [−0.470–0.015]) (males -females).

MMM analysis with backward selection of significant predictors, resulted in a model including sex and side as significant variables. In this final model the adjusted difference between PD patients and controls was not significant (t(78) =1.786, *p* = .078, d = 0.218, 95% CI [−0.025, 0.461] (patients-controls).

## 4 Discussion

In this cross-sectional study, we show that the CSA of the vagus nerve is not different in PD patients compared to controls. In addition, no relation between the CSA of the vagus nerve and autonomic symptoms as assessed by the SCOPA-AUT questionnaire was found. The CSA was neither influenced by age, sex, diabetes mellitus and cardiovascular disease. Consistent with previous studies, the CSA of the right vagus nerve was significantly larger in both the PD and control group [[16], [17], [18], [19], [20],[26], [27], [28]].

Our findings confirm the findings of two studies which also found no reduction in CSA of the vagus nerve in PD patients [20,21]. We were unable to replicate the reduction in CSA as reported by earlier studies [[17], [18], [19]]. Important to note is the high variation in CSA measured between studies. The mean CSA in control participants varies from 1.3 to 2.7 mm^2^ for the right and 1.1 to 2.6 for the left vagus nerve, this is in line with recent published systematic review and meta-analysis [26]. In the PD groups the variation is even larger between studies. In our cohort we also found a high variance in CSA between individuals.

The validity of our findings is supported by several factors. First, we have shown a high inter-rater reliability. Second, our method (drawing a circle within the epineural rim to determine the CSA) has a high correlation with previously used method, in which the longest cross-sectional diameter *a* and the diameter *b* perpendicular to *a* were used to calculate the CSA [19]. Third, our findings replicate the well-known right-left difference of the CSA of the vagus nerve [[16], [17], [18], [19], [20],[26], [27], [28]]. This right-left difference in CSA is explained by the different functional anatomy of the vagus nerve. The right vagus nerve innervates parts of the small intestine, the colon and also a part of the gastric plexus. The left vagus nuclei terminate in the anterior plexus and from there on new branches go to the stomach, liver and the superior part of the abdomen. Fourth, the range of the CSA of the vagus nerve we found is similar to previous reports [17,20,26,27].

A possible reason why in our study no difference was found between PD and controls is the low prevalence of autonomic symptoms in our PD group. The mean score at the SCOPA-AUT for Parkinson patients is reported to be higher in other studies [23]. It could be postulated that patients with more autonomic symptoms might show a reduced CSA of the vagus nerve. This could make the vagal nerve ultrasound a useful screening tool to detect autonomic dysfunction, but not to diagnose persons with PD in an early stage of disease or even in a pre-symptomatic phase. On the other hand, our analysis did not show any relation between the autonomic symptoms and the CSA of the vagus nerve. Other groups, however, have observed a clear relation between autonomic symptoms and the CSA of the vagal nerve using the NMS-QUEST and assessment of heart rate variability [17,29]. There might be several reasons for the discrepancy. First, our PD group did score relatively low on the SCOPA-AUT. Next, the NMS-quest and use of heart rate variability might be better tools to assess autonomic dysfunction. One could argue that our control group was not a completely healthy population.

### 4.1 Limitations

The control group was recruited from a group with a higher risk on cardiovascular comorbidities. This could however also be considered as a strength as one of the differential diagnoses of the idiopathic form of PD is vascular parkinsonism. Another limitation of our study is that also in de PD group some patients had diabetes mellitus or cardiovascular diseases. As atrophy of the vagus nerve has also been reported in patients with diabetes mellitus. On the other hand, a reliable diagnostic tool to diagnose PD should be able to differentiate between patients suffering from PD and person with a higher risk on cardiovascular complications or diabetes mellitus. Besides, we showed with the mixed model analysis that there is no correlation between cardiovascular diseases or diabetes mellitus and CSA of the vagus nerve in our cohort. Another limitation of our study is that the sonographer was not blinded for the diagnosis. It thus remains a challenging search to find a reliable and cheap biomarker in the early or preclinical diagnosis of PD. Considering the high interindividual variance in the CSA a reduction over time per individual might be able to provide a better prediction on the risk of developing PD. We would like to emphasize that the findings of our study and other studies published are not congruent. A possible reason for this could be that confounding effects of certain PD subtypes, disease severity as well as the appearance of autonomic symptoms exist. We argue that a larger (multicenter) study is warranted to address these questions.

## 5 Conclusion

The CSA of the vagus nerve can be measured with high accuracy. It is not reduced in PD patients and is neither correlated with autonomic symptoms, sex nor age. Therefore, we conclude that the results of this study do not support that a single ultrasound examination of the vagus nerve is at this moment a suitable biomarker in the diagnosis of PD.

## CRediT authorship contribution statement

**Laura C.J. Sijben:** Conceptualization, Data curation, Formal analysis, Investigation, Methodology, Project administration, Resources, Validation, Visualization, Writing – original draft, Writing – review & editing. **Werner H. Mess:** Conceptualization, Data curation, Supervision, Visualization, Writing – review & editing. **Uwe Walter:** Conceptualization, Visualization, Writing – review & editing. **A. Miranda L. Janssen:** Formal analysis, Methodology, Validation, Visualization, Writing – review & editing. **Mark Kuijf:** Resources, Writing – review & editing. **Mayke Oosterloo:** Resources, Writing – review & editing. **Wim M. Weber:** Resources, Writing – review & editing. **Marcus L.F. Janssen:** Conceptualization, Data curation, Formal analysis, Investigation, Methodology, Project administration, Supervision, Resources, Validation, Visualization, Writing – original draft, Writing – review & editing.
